# Improving Energy Storage Properties of Barium Zirconate Titanate Ceramics via Defect Dipole Engineering

**DOI:** 10.3390/ma18122809

**Published:** 2025-06-15

**Authors:** Zhiyi Wang, Zhengchao Qin, Si Gao, Hongjuan Zheng, Jin Luo, Yunfei Liu, Yinong Lyu

**Affiliations:** 1 The State Key Laboratory of Materials-Oriented Chemical Engineering, College of Materials Science and Engineering, Nanjing Tech University, Nanjing 210009, China; zywang@njtech.edu.cn (Z.W.); zcqin@njtech.edu.cn (Z.Q.); zhenghj@njtech.edu.cn (H.Z.); jluuky2014@njtech.edu.cn (J.L.); yfliu@njtech.edu.cn (Y.L.); yinonglu@njtech.edu (Y.L.); 2 Jiangsu National Synergetic Innovation Center for Advanced Materials (SICAM), Nanjing 210009, China; 3 Jiangsu Collaborative Innovation Center for Advanced Inorganic Function Composites, Nanjing 210009, China

**Keywords:** ferroelectric, ceramics, BZT, defect dipoles

## Abstract

Lead-free ceramic materials have been widely studied since dielectric capacitors became a key component for energy storage. In this work, we adopted defect dipole engineering and improved the energy storage performance of barium zirconate titanate (BZT) ceramics by doping them with MnO_2_. With the increase in Mn content, the hysteresis loop changed from a conventional loop to a pinned hysteresis loop, resulting in a decrease in remnant polarization (*P*_r_). When x = 0.02, the recoverable energy storage density (*W*_rec_) reached 0.1561 J/cm^2^ @ 40 kV/cm, a 59% increase from undoped BZT. Further, XPS and EPR analyses confirmed that many oxygen vacancies were generated. We also performed SEM and TEM characterization and observed the microstructures. These results are consistent with theories suggesting that the formation of the pinned hysteresis loop is attributable to oxygen vacancies and defect dipoles.

## 1. Introduction

With the widespread use of renewable clean energy, energy storage is becoming increasingly important. Dielectric capacitors have become a key component for energy storage due to their ultra-high power density and fast charging−discharging rates. At present, lead zirconate titanate-based ceramics are widely used in industrial applications. However, lead may pose a threat to human health and the environment due to its toxicity [1,2,3,4]. Therefore, in recent years, a series of lead-free ceramics have been developed and studied. Barium titanate is one of the most widely used lead-free materials, and due to its perovskite structure, it features high polarization, low loss, and is commonly used in the manufacturing of multilayer ceramic capacitors. In addition, barium titanate (BT) has superior properties such as higher chemical stability and a wider operating temperature range. Ge P Z et al. [5] achieved excellent *W*_rec_ = 3.62 J/cm^2^ and η = 88.5% in 0.88BT-0.12BZH ceramics by introducing BZH into BT under an electric field of 240 kV/cm. Therefore, the structure of its BT-based ceramics is adjustable, and the influence of different doped ions or solid solutions on its performance is different. In replacing Ti^4+^ with Zr^4+^ to form a barium zirconate titanate (BZT) solid solution, it has been shown that the compound has excellent dielectric and ferroelectric properties. However, its relatively high residual polarization (*P*_r_) limits its application in energy storage devices [6,7,8,9,10].

The key performance of capacitors for energy storage use is both the recoverable energy storage density (*W*_rec_) and energy storage efficiency (*η*). The most widely used methods in the last decade have been compositional solid substitution and building a morphotropic phase boundary (MPB). Zr^4+^, Hf^4+,^ and Sn^4+^ are the most utilized cations for B-site substitution in BT-based ceramics, as these elements usually take a large atomic percent and form a solid perovskite structure. For instance, when the atomic percentage of Zr^4+^ exceeds 20%, the ceramics exhibit a diffused phase transition at room temperature. However, when the atomic percentage of Zr^4+^ continually increases, the hysteresis loop becomes more “slim”, but the maximum polarization (*P*_max_) decreases [11]. Another way to improve energy storage performance is to change the “shape” of the hysteresis loop. For example, antiferroelectric materials exhibit double hysteresis loops, displaying extremely low residual polarization (*P*_r_), a low coercive field (*E*_c_), and moderately low dielectric losses. Therefore, double hysteresis loops can achieve excellent energy storage properties. *P*_max_ is relatively high, compared with relaxor ferroelectrics. However, there are still many challenges in designing double hysteresis loops for BT-based ceramics without aging [12].

**Figure 1:**
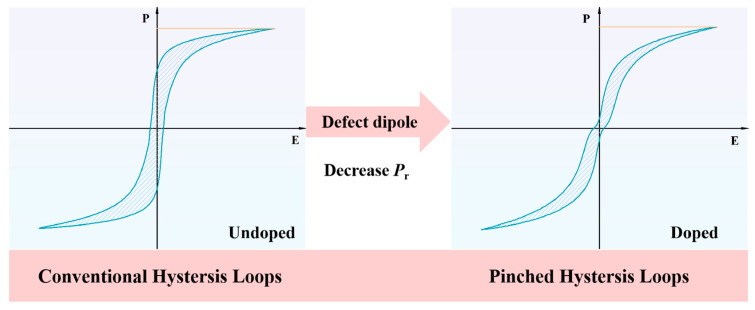
Diagram of the defect dipole engineering.

With the development of ferroelectric materials, it has generally been accepted that a double hysteresis loop is derived from an antiferroelectric material. For example, by hard doping or acceptor doping, i.e., replacing high-valence cations in ferroelectric materials with low-valence metal cations, oxygen vacancies are generated to maintain the electrical neutrality of the unit cell. Oxygen vacancies and acceptors form defect dipoles, making domain walls difficult to move. Loops similar to double hysteresis loops are often referred to as contraction hysteresis loops [13]. In recent years, many studies have obtained similar results through hard doping. In ceramics, the defect dipole formed by oxygen vacancies combined with doped ions (such as Fe^3+^, Mn^3+^, etc.) has an important effect on the dielectric relaxation, conductivity, and ferroelectric properties of materials [13,14]. As shown in Figure 1, the pinched hysteresis loops can lead to a dramatic decrease in *P*_r_, ultimately resulting in an improvement in energy storage properties [15,16].

In this work, we adopted the design concept of defect dipole engineering and doped BaZr_0_._07_Ti_0_._93_O_3_ ceramics with MnO_2_. The energy storage properties of the material were enhanced by the generated pinched hysteresis loop and compared with undoped BZT ceramics. Further, we utilized XPS, EPR, SEM, and TEM to investigate the effect of defect dipoles on microstructures. Hence, this work can be a reference for defect dipole engineering and observation of defect dipoles.

## 2. Materials and Methods

### 2.1. Solid-State Synthesis

The basic methods for synthesizing BZT ceramics include the traditional solid-phase, sol–gel, and hydrothermal methods. These methods synthesize target materials by directly reacting with solid raw materials at high temperatures. BaZr_0_._07_Ti_0_._93-x_O_3_-xmolMnO_2_ (BZT-xMn) ferroelectric ceramics were prepared using the conventional solid-state method. Barium carbonate (BaCO_3_, 99%), zirconium dioxide (ZrO_2_, 99%), titanium dioxide (TiO_2_, 99%), and manganese dioxide (MnO_2_) were used as raw materials. The mixture was wet-ball-milled for 12 h with anhydrous ethanol, fully dried, and then calcined at 1200 °C for 5 h. The calcined powders were mixed with 7% polyvinyl alcohol (PVA) and pressed at 10 MPa to produce 13 mm diameter pellets. These ceramic pellets were sintered at 1400 °C in air for 3 h and then cooled down in a furnace. In order to observe the surface morphology of ceramics through SEM, the sintered ceramics were ground and polished with emery of different mesh sizes before being cleaned with an ultrasonic cleaner. After being dried in an oven, these ceramic samples were placed in a JFC-1600 ion sputtering instrument and underwent gold spraying treatment for approximately 90 s in a vacuum environment. Then, the sample was placed in the scanning electron microscope. To observe the microstructure of the sample, the polished ceramic plate was first adhered to the glass plate. Then, it was ground with emery to a thickness of about 50 microns. Finally, after ultrasonic cleaning and drying, it was placed in a transmission electron microscope for testing.

### 2.2. Characterization

The crystal structure of the ceramics was characterized by an X-ray diffractometer (XRD, Rigaku Corporation, Tokyo, Japan). The microstructure of the ceramics was characterized using a scanning electron microscope (SEM, JSM-6510, Tokyo, Japan) and a transmission electron microscope (TEM, JEM-2100F, JEOL, Tokyo, Japan). Dielectric properties of the ceramics were measured with an impedance analyzer (4294A, Agilent, Santa Clara, CA, USA). Hysteresis loops were tested using a ferroelectric analyzer (Precision Premier II, Radiant Technologies, Los Angeles, CA, USA). X-ray photoelectron spectrum was performed using a Thermofisher spectrometer (XPS, Nexsa, Thermofisher, Waltham, MA, USA). The electron paramagnetic resonance spectrum was measured using a Bruker spectrometer (EMXplus-6/1, Bruker, Billerica, MA, USA).

## 3. Results and Discussion

### 3.1. Ferroelectric and Dielectric Properties

**Figure 2:**
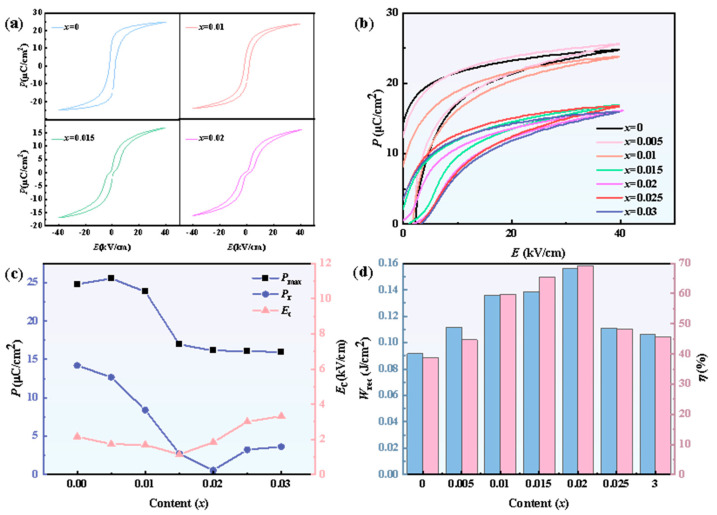
(**a**) Hysteresis loops for BZT-xMn (x = 0, 0.01, 0.015, 0.02); (**b**) unipolar hysteresis loop diagram for BZT-xMn (x = 0 − 0.03); (**c**) line chart of *E*_c_, *P*_r_, and *P*_max_ with corresponding x content; (**d**) histogram of recoverable energy storage density (*W*_rec_) and energy storage efficiency (*η*).

As illustrated in Figure 2a, due to the increase in the Mn component, the hysteresis loop changed from a conventional loop to a pinched hysteresis loop. This result is consistent with our expectations since we adopted the defect dipole engineering method. For comparison, the unipolar hysteresis loops of all Mn components (x = 0–0.03) are shown in Figure 2b, while *E*_c_, *P*_r_, and *P*_max_ are extracted and shown in Figure 2c. When x increased to 0.01, we found a significant decrease in *P*_r_ while *P*_max_ did not change very much. When x increased to 0.015, both *P*_r_ and *P*_max_ decreased. However, when x was above 0.02, *P*_max_ remained unchanged while *E*_c_ and *P*_r_ demonstrated small increases. These changes led to the variation in the energy storage properties. These energy storage properties can be calculated using Equations (1)–(3) and are shown in Figure 2d:

(1) $$W_{d}=\int_{0}^{P_{max}}EdP,$$

(2) $$W_{rec}=\int_{P_{r}}^{P_{max}}EdP,$$

(3) $$\eta =\frac{W_{rec}}{W_{d}}\times 100\%,$$

where *W*_d_ and *W*_rec_ stand for total charge energy density and recoverable (discharge) energy storage density, respectively. E is the applied electric field. When x = 0.02, *W*_rec_ = 0.1561 J/cm^2^ @ 40 kV/cm and η = 69.21%. Both properties reach the maximum value, with the *W*_rec_ value demonstrating a 59% increase compared with that of pure BaZr_0_._07_Ti_0_._93_O_3_ without Mn doping.

Although energy storage properties have improved, challenges remain for further improvement. One of these challenges is reducing *P*_max_, which has been observed in many similar cases where defect dipoles were induced [17]. There are many explanations for the pinched hysteresis loops phenomenon, such as residual internal stress models and space charge models. Ren et al. [18,19] proposed that the defect dipole polarization cannot switch quickly with an external electric field. When the external electric field weakens, the “pinched” defect dipole polarization provides a restorting force for the domains to switch back to their initial states. This leads to the macroscopic appearance of the pinched hysteresis loops phenomenon. This model has been widely accepted. In 2017, Liu et al. [20] provided a first-principles-based atomistic model and quantitatively explained the relationship between hysteresis loops and defect dipoles. Continuous attention has been paid to defect dipoles; however, many questions remain unsolved. Our current understanding of defect dipoles and the “pinning” effect is mostly limited to the domain level. Detailed atomistic mechanisms are still lacking, both theoretically and experimentally [21,22,23].

**Figure 3:**
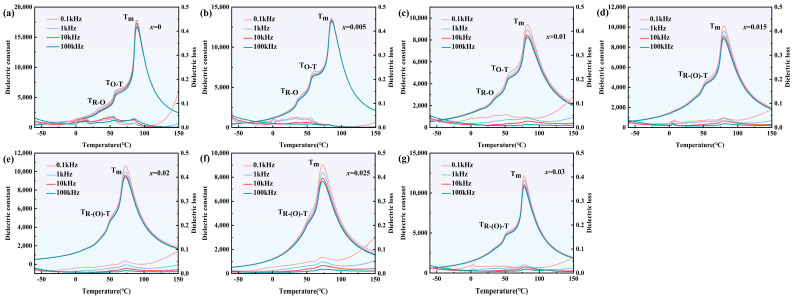
Temperature-dependent dielectric constant and loss of BZT-xMn ceramics. (**a**) x = 0; (**b**) x = 0.005; (**c**) x = 0.01; (**d**) x = 0.015; (**e**) x = 0.2; (**f**) x = 0.025; (**g**) x = 0.03.

Temperature-dependent dielectric constant and loss of BZT-xMn (x = 0–0.03) ceramics are shown in Figure 3a–g. With the increase in x, T_R-O_ becomes high-temperature, while T_O-T_ and T_m_ slowly become low-temperature. T_R-O_, T_O-T,_ and T_m_ represent rhombohedral–orthorhombic (R-O), orthorhombic–tetragonal (O-T), and tetragonal–cubic (T-C) transition temperatures, respectively. When x ≥ 0.015, T_R-O_ can hardly be observed. This phenomenon is similar to that of Zr content [24,25]. With increased Zr content, T_R-O_ and T_O-T_ gradually merge as a single rhombohedral–tetragonal (R-T) phase transition. In our cases, when x ≥ 0.015, the curves became smooth, and only one prominent transition peak can be seen, which means that the R-O transition somehow becomes diffused at room temperature. The remaining phase transition was thus labeled R-(O)-T transition. Meanwhile, we noticed that the dielectric losses were small across all spectra. However, the dielectric losses at low frequencies were relatively larger, indicating the existence of point defects, as the relationship between defects and low-frequency dielectric response has been studied [26,27].

### 3.2. Defect Characterization

**Figure 4:**
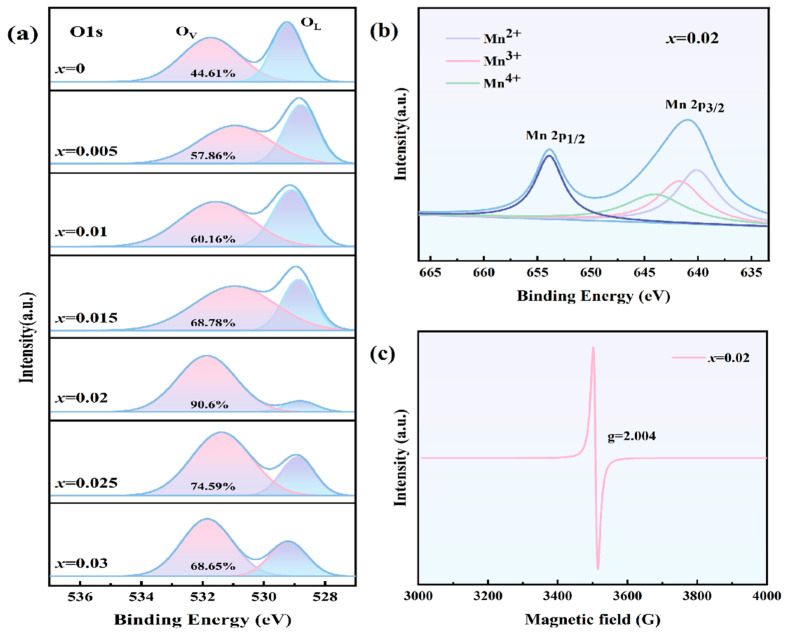
(**a**) XPS spectra of O1s orbitals for BZT-xMn (x = 0–0.03) ceramics; (**b**) XPS spectra of Mn2p orbitals when x = 0.02 (The purple line represents Mn^2+^, The pink line represents Mn^3+^ and The green line represents Mn^4+^); (**c**) EPR spectra of BZT-xMn ceramics when x = 0.02.

XPS characterization was carried out to study the mechanism of tightening the hysteresis loop. The spectra of O1s orbitals for BZT-xMn (x = 0–0.03) ceramics are displayed in Figure 4a. It was generally believed that the peak around 532 eV represents an unstable surface that generates surface hydroxyls [28]. However, many studies have confirmed that the peak around 532 eV coincides with oxygen vacancies [29,30,31]. Meanwhile, the peak below 530 eV is the oxygen found in the stable perovskite structure. The changing ratio between the two oxygen peaks indicates that with the increase in Mn doping, a large amount of oxygen vacancies are generated. This also coincides with the EPR results shown in Figure 4c. The peak with g = 2.004 also indicated the existence of oxygen vacancies [32,33]. Specifically, the vacancy oxygen percentage reached 44.61%, 57.86%, 60.16%, 68.78%, 90.6%, 74.59%, and 68.65% when the Mn dopant increased from 0.005 to 0.03. We did not notice any linear increases in vacancies with dopant concentration. As the Mn content increased, the oxygen vacancies also increased and then decreased. This is because at low doping concentrations, oxygen vacancies increase to compensate for the induced charge. When the doping concentration reaches a certain level, defect dipoles are formed, which inhibit the further increase of oxygen vacancies [34]. Much research has been conducted, and many studies have focused on oxygen vacancies, while many novel properties are believed to be generated from oxygen vacancies. Currently, we know that oxygen vacancies are always present in perovskite materials [35]. Hence, oxygen vacancies may vary between samples. However, deeper reasons for the formation and existence of oxygen vacancies still need further study.

Defect dipoles are generated from both the Mn dopant and oxygen vacancies (Vö), as confirmed by a series of studies [18]. Chikada et al. [36] further found that the valence state of Mn changes according to the Vö density by first-principles calculations and EPR measurement. In other words, Mn dopant and oxygen vacancies form defect dipole [Mn_Ti/Zr_’’-Vö] and [2Mn_Ti/Zr_’-Vö]. This finding aligns with the Mn 2p orbital XPS measurement shown in Figure 4b. Through Mn 2p3/2 orbital multi-peak fitting, the three Mn valence states, namely Mn^4+^ (644.1 eV), Mn^3+^ (641.7 eV), and Mn^2+^ (640.1 eV), coexist [37,38].

### 3.3. Microstructure Characterization

**Figure 5:**
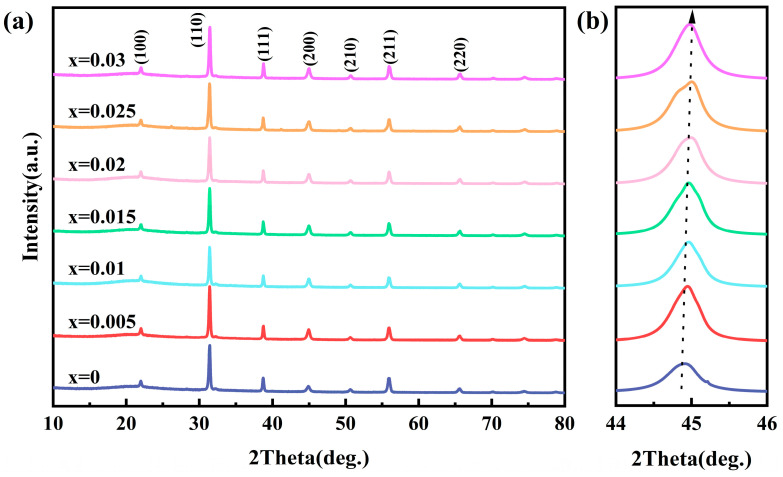
(**a**) XRD pattern of BZT-xMn (x = 0 − 0.03) ceramics; (**b**) Enlarged image at 44-46 degrees.

The room-temperature XRD of BZT-xMn (x = 0–0.03) ceramics with different Mn contents is shown in Figure 5. With the increase in x, all components present a standard perovskite structure without forming a second phase, indicating that all Mn has entered the BZT lattice and all components have formed a single solid solution. With the increase in x, the peak near 45° gradually shifts to the right due to the substitution of polyvalent Mn ions for smaller Ti^4+^/Zr^4+^; consequently, the cell shrinks. Thus, the crystal plane spacing for the 110 crystal orientation, which represents the strongest diffraction peak, is derived. The crystal plane spacing is d = 2.8466 when x = 0–0.02, and when x = 0.025, d = 2.8467, and when x = 0.03, d = 2.8448.

**Figure 6:**
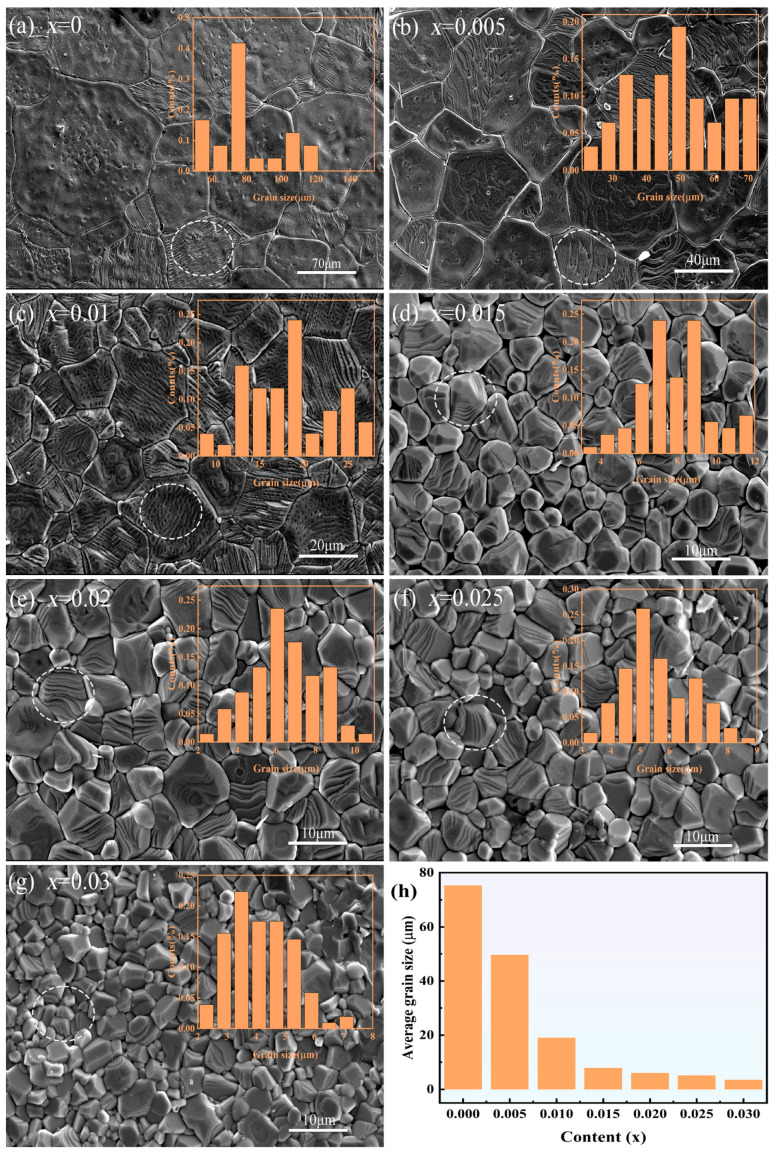
Secondary electron images of BaZr_0_._07_Ti_0_._93-x_O_3_–xMnO_2_ ceramics(The white circles represent macroscopic domain structures). (**a**) x = 0; (**b**) x = 0.005; (**c**) x = 0.01; (**d**) x = 0.015; (**e**) x = 0.02; (**f**) x = 0.025; (**g**) x = 0.03; (**h**) A point-line graph of the average grain size varying with x.

Ferroelectrics are characterized by ferroelectric domains, and their properties are mainly controlled by ferroelectric domain structures and their evolutions, including the motion of domain walls. Thus, the study of ferroelectric domain structures is of both fundamental interest and technological importance and is expected to help deepen the understanding of ferroelectric property variations in BT-based ceramics. Figure 6a–g show secondary electron images in a scanning electron microscope (SEM). All samples exhibit low porosity. With the increase in x, it can be observed that the grain size also decreased, from about 75 mm to 5 mm, indicating that excessive MnO_2_ inhibits the growth of grains [39,40]. Grain size statistics are given in the upper right corners of each corresponding figure, and the line graph for the average grain size is displayed in Figure 6h. In addition, for x = 0, 0.005, 0.01, we can see ripple-like domains, as shown in the dashed circle in Figure 6a–c, respectively. However, with the increase in x, the density of domains becomes much sparser, as shown in Figure 6d–g.

**Figure 7:**
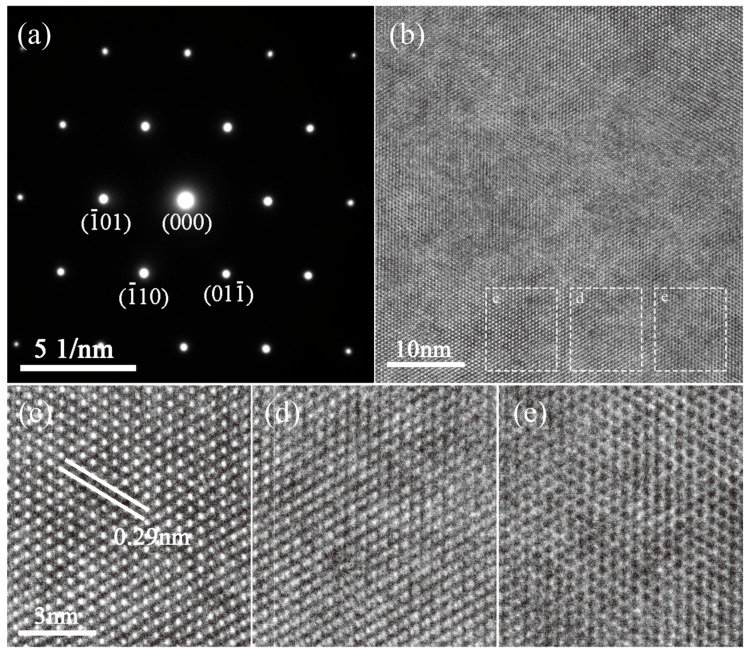
(**a**) Axial selected area electron diffraction (SAED) at x = 0.02 in [111]; (**b**) high-resolution transmission electron microscope (HRTEM) image; (**c**–**e**) Enlarged image from the dash box in Figure 7b.

Figure 7a shows the selected area diffraction pattern (SAED) of the BaZr_0_._07_Ti_0_._93-x_O_3_-0.02MnO_2_ ceramic with the [111] zone axis. Figure 7b shows the correspond high-resolution transmission electron microscope (HRTEM) image of the sample, while the enlarged images, as shown in Figure 7b–d, are cut from the dashed boxes in Figure 7b, respectively. However, the contrast between Figure 7b,d is quite different, indicating that the zone axis varies between these regions. Lattice spacing can be obtained directed from the images, and the lattice spacing is deemed to be 0.29 nm, as illustrated in Figure 7c. The spacing is consistent with both the SAED and XRD results.

## 4. Conclusions

In summary, we adopted defect dipole engineering and improved the energy storage performance of BZT ceramics by doping with MnO_2_. With the increase in Mn content, the hysteresis loop changes from a conventional loop to a pinned hysteresis loop, resulting in a decrease in *P*_r_ and an improvement in recoverable energy storage density and efficiency. XPS and EPR analyses confirmed that many oxygen vacancies were generated with Mn doping and formed defect dipoles. Furthermore, we also performed SEM and TEM characterization and observed the microstructures. Based on the XPS results, these defects are formed due to oxygen vacancies and defect dipoles. These defects pinched the movement of domain walls and formed a pinched hysteresis loop, which is consistent with both theories and our expectations.

## Data Availability

The original contributions presented in this study are included in the article. Further inquiries can be directed to the corresponding author.

## References

[1] Wang G., Lu Z., Li Y., Li L., Ji H., Feteira A., Zhou D., Wang D., Zhang S., Reaney I.M. (2021). Electroceramics for high-energy density capacitors: Current status and future perspectives. Chem. Rev..

[2] Zheng M., Hou Y., Zhu M., Zhang M., Yan H. (2014). Shift of morphotropic phase boundary in high-performance fine-grained PZN–PZT ceramics. J. Eur. Ceram. Soc..

[3] Kour P., Pradhan S.K., Kumar P., Sinha S.K., Kar M. (2016). Enhanced ferroelectric and piezoelectric properties in La-modified PZT ceramics. Appl. Phys. A.

[4] Thong H.-C., Zhao C., Zhou Z., Wu C.-F., Liu Y.-X., Du Z.-Z., Li J.-F., Gong W., Wang K. (2019). Technology transfer of lead-free (K,Na)NbO_3_-based piezoelectric ceramics. Mater. Today.

[5] Ge P.-Z., Liu Z.-G., Huang X.-X., Tang X.-G., Tang Z.-H., Li S.-F., Liu Q.-X., Jiang Y.-P., Guo X.-B. (2023). Excellent energy storage properties realized in novel BaTiO_3_-based lead-free ceramics by regulating relaxation behavior. J. Mater..

[6] Yan F., Qian J., Wang S., Zhai J. (2024). Progress and outlook on lead-free ceramics for energy storage applications. Nano Energy.

[7] Li Y., Tang M.-Y., Zhang Z.-G., Li Q., Li J.-L., Xu Z., Liu G., Li F. (2023). BaTiO_3_-based ceramics with high energy storage density. Rare Met..

[8] Chen Y., Fan H., Hou D., Jia Y., Zhang A., Wang W. (2022). Bismuth sodium titanate-barium titanate-barium zirconate titanate relaxor ferroelectric ceramics with high recoverable energy storage density. Ceram. Int..

[9] Yang H., Bao W., Lu Z., Li L., Ji H., Huang Y., Xu F., Wang G., Wang D. (2021). High-energy storage performance in BaTiO_3_-based lead-free multilayer ceramic capacitors. J. Mater. Res..

[10] Dai Z., Xie J., Liu W., Wang X., Zhang L., Zhou Z., Li J., Ren X. (2020). Effective strategy to achieve excellent energy storage properties in lead-free BaTiO_3_-based bulk ceramics. ACS Appl. Mater. Interfaces.

[11] Tian Y., Xue F., Li W., Gan Y., Zheng Y., Du G. (2025). Direct measurement of large electrocaloric effect in BZT-BST lead-free relaxor ferroelectrics near room temperature. Ceram. Int..

[12] Jin L., Li F., Zhang S. (2014). Decoding the Fingerprint of ferroelectric loops: Comprehension of the material properties and structures. J. Am. Ceram. Soc..

[13] Zhao C., Wang Y., Li Z., Chen W., Xu Q., He D., Xi D., Zhang Q., Yuan T., Qu Y. (2019). Solid-diffusion synthesis of single-atom catalysts directly from bulk metal for efficient CO_2_ reduction. Joule.

[14] Lin J., Qian J., Shi Y., Wang S., Lin J., Ge G., Hua Y., Shen B., Zhai J. (2024). Enhanced piezoelectric response attained by defect dipoles in BiFeO_3_-based lead-free ceramics. ACS Appl. Mater. Interfaces.

[15] Fu W., Lu Y.-q., Han Q., Hu T.-Y., Duan T., Liu Y., Cheng S.-D., Dai Y., Liu M., Ma C. (2025). Enhanced energy storage performance of 0.85BaTiO_3_–0.15Bi(Mg_0.5_Hf_0.5_)O_3_ films via synergistic effect of defect dipole and oxygen vacancy engineering. Acta Mater..

[16] Kim J., Saremi S., Acharya M., Velarde G., Parsonnet E., Donahue P., Qualls A., Garcia D., Martin L.W. (2020). Ultrahigh capacitive energy density in ion-bombarded relaxor ferroelectric films. Science.

[17] Wang H.Q., Dai Y.J., Zhang X.W. (2012). Microstructure and hardening mechanism of K_0.5_Na_0.5_NbO_3_ Lead-Free Ceramics with CuO Doping Sintered in Different Atmospheres. J. Am. Ceram. Soc..

[18] Zhang L.X., Ren X. (2005). In situ observation of reversible domain switching in aged Mn-doped BaTiO_3_ single crystals. Phys. Rev. B.

[19] Ren X., Otsuka K. (2000). Universal symmetry property of point defects in crystals. Phys. Rev. Lett..

[20] Liu S., Cohen R.E. (2017). Multiscale simulations of defect dipole–enhanced electromechanical coupling at dilute defect concentrations. Appl. Phys. Lett..

[21] Zhang Y., Li A., Zhang G., Zheng Y., Zheng A., Luo G., Tu R., Sun Y., Zhang J., Shen Q. (2022). Optimization of energy storage properties in lead-free barium titanate-based ceramics via B-site defect dipole engineering. ACS Sustain. Chem. Eng..

[22] Feng Y., Wu J., Chi Q., Li W., Yu Y., Fei W. (2020). Defects and aliovalent doping engineering in electroceramics. Chem. Rev..

[23] Zhao Z.-H., Dai Y., Huang F. (2019). The formation and effect of defect dipoles in lead-free piezoelectric ceramics: A review. Sustain. Mater. Technol..

[24] Peng J., Shan D., Liu Y., Pan K., Lei C., He N., Zhang Z., Yang Q.J. (2018). A thermodynamic potential for barium zirconate titanate solid solutions. Npj Comput. Mater..

[25] Maiti T., Guo R., Bhalla A.S. (2008). Structure-property phase diagram of BaZr_x_Ti_1−x_O_3_ System. J. Am. Ceram. Soc..

[26] Sundarakannan B., Kakimoto K., Ohsato H.J.J.o.A.P. (2003). Frequency and temperature dependent dielectric and conductivity behavior of KNbO_3_ ceramics. J. Appl. Phys..

[27] Prieto C., Arizmendi L., Gonzalo J.A., Jaque F., Agulló-López F. (1986). Point-defect contribution to the low-frequency dielectric response of LiTaO_3_ Reanalysis. Phys. Rev. B.

[28] Frankcombe T.J., Liu Y. (2023). Interpretation of oxygen 1s X-ray photoelectron spectroscopy of ZnO. Chem. Mater..

[29] Shi Z., Tong S., Wei J., Guo Y., Zhang Y., Wang L., Zhang J. (2022). Regulating Multiscale Defects to enhance the thermoelectric performance of Ca_0.87_Ag_0.1_Dy_0.03_MnO_3_ ceramics. ACS Appl. Mater. Interfaces.

[30] Liu J., Zhang D., Yan Y., Li Z., Li F., Yang S. (2024). Piezoelectric ceramic hardening through defect distribution optimization in multicomponent systems. Adv. Funct. Mater..

[31] Sun Z., Bai Y., Jing H., Hu T., Du K., Guo Q., Gao P., Tian Y., Ma C., Liu M. (2024). A polarization double-enhancement strategy to achieve super low energy consumption with ultra-high energy storage capacity in BCZT-based relaxor ferroelectrics. Mater. Horiz..

[32] Zhang Q., Tang J., Du P., Li W., Yuan G., Liu Z., Luo L. (2021). Reversible and color controllable emissions in Er^3+^/Pr^3+^-codoped K_0.5_Na_0.5_NbO_3_ ceramics with splendid photochromic properties for anti-counterfeiting applications. J. Eur. Ceram. Soc..

[33] Zhang Z., Chen L., Luo H., Zhang Y., Yao Y., Liu G., Qi H., Chen J. (2023). Outstanding electromechanical performance in piezoelectric ceramics via synergistic effects of defect engineering and domain miniaturization. J. Mater. Sci. Technol..

[34] Li X., Zhu L., Huang P., Chen Z., Bai W., Li L., Wen F., Zheng P., Wu W., Zheng L. (2020). Reduction of oxygen vacancy concentration and large enhancement of electrical performances in Cu/Sb co-doped Bi_4_Ti_3_O_12_ high temperature piezoelectric ceramics. J. Appl. Phys..

[35] Ji Q., Bi L., Zhang J., Cao H., Zhao X.S. (2020). The role of oxygen vacancies of ABO_3_ perovskite oxides in the oxygen reduction reaction. Energy Environ. Sci..

[36] Chikada S., Kubota T., Honda A., Higai S.I., Motoyoshi Y., Wada N., Shiratsuyu K. (2016). Interactions between Mn dopant and oxygen vacancy for insulation performance of BaTiO_3_. J. Appl. Phys..

[37] Guo Y.Y., Zhao Y., Zhang H.G., Zhang N. (2017). Aging-induced hysteresis loop evolution of Ba(Ti_0.99_Mn_0.01_)O_3_ ceramics during ferroelectric-ferroelectric transition cycle. J. Alloys Compd..

[38] Yin Y., Yu J.-R., Tang Y.-C., Song A.-Z., Liu H., Yang D., Li J.-F., Zhao L., Zhang B.-P. (2022). Enhanced energy storage properties and antiferroelectric stability of Mn-doped NaNbO_3_-CaHfO_3_ lead-free ceramics: Regulating phase structure and tolerance factor. J. Mater..

[39] Shang F., Wei J., Xu J., Zhang H., Xia Y., Zhu G., Jiang K., Chen G., Ye Z., Xu H. (2023). Boosting energy storage performance of glass ceramics via modulating defect formation during crystallization. Adv. Sci..

[40] Zhang F., Tan J., Wang P., Huang R., Lin H.-T., Huang X., Yang J., Fu Z., Cao X., Zhang L. (2024). Defect dipole engineering enhanced the dielectric performance and reliability of Mn-doped BaTiO_3_-based multilayer ceramic capacitor. Ceram. Int..

